# A systematic review reveals that African children of 15–17 years demonstrate low hepatitis B vaccine seroprotection rates

**DOI:** 10.1038/s41598-023-49674-1

**Published:** 2023-12-13

**Authors:** Fahad Muwanda, Hakim Sendagire, Gerald Mboowa, David Patrick Kateete, Beatrice Achan, Ezekiel Mupere, Hussein Mukasa Kafeero, Bernard Ssentalo Bagaya

**Affiliations:** 1 Department of Medical Microbiology, Faculty of Health Sciences, Habib Medical School, Islamic University in Uganda, P.O. Box 7689, Kampala, Uganda; 2 Department of Immunology and Molecular Biology, School of Biomedical Sciences, College of Health Sciences, Makerere University, P.O. Box 7072, Kampala, Uganda; 3 Department of Medical Microbiology, School of Biomedical Sciences, College of Health Sciences, Makerere University, P.O. Box 7072, Kampala, Uganda; 4 Department of Paediatrics and Child Health, School of Medicine, College of Health Sciences, Makerere University, P.O. Box 7072, Kampala, Uganda; 5 The African Center of Excellence in Bioinformatics and Data-Intensive Sciences, Infectious Diseases Institute, College of Health Sciences, Makerere University, P.O. Box 22418, Kampala, Uganda

**Keywords:** Immunology, Health care

## Abstract

Childhood HBV immunization remains globally fundamental to the elimination of hepatitis B virus (HBV). However, monitoring proportions of HBV vaccine seroprotection and their determinants among African Pediatric recipients is crucial. This study sought to verify extent of immune protection accorded by the HBV vaccine in African children of up to 17 years of age by pooling the prevalence of seroprotection reported by primary studies conducted in the Northern, Western, and Southern African regions. We included 19 eligible articles out of the 197 initially downloaded, published from 1999 to 2021 from African Journals Online (AJOL), EMBASE, Scopus, and PubMed. The study protocol was registered with the International Prospective Register of Systematic Reviews (PROSPERO), University of York Centre for Reviews and Dissemination, under the registration number CRD42022361277. Significantly higher (*p* < 0.0001) proportion of HBV vaccine seroprotection (69.07%) was found among children under 15 years of age than children 15–17 years (32.368%), 95% CI [34.2454–39.0847%]. Whereas successful integration of the HBV vaccine on the extended programs on immunizations (EPI) has been a major achievement in the reduction of HBV infection in Africa, markedly reduced HBV vaccine seroprotection is persistently demonstrated among adolescent children 15–17 years of age. Future studies are required to clarify the need for booster dose vaccination in most at risk populations and age groups.

## Introduction

Hepatitis B virus (HBV) infection is still a major global public health problem, with 292 million people infected, translating into a global prevalence of 3.9%^1^. Although universal vaccination had decreased prevalence of HBV in children younger than 5 years from 4.7% in the pre-vaccination era (1980s to early 2000s) to 1.3% by 2015, prevalence in the WHO Africa region remains at 3%. Sub-Saharan Africa is disproportionately over-burdened^2^. In highly endemic countries like Uganda, HBV national prevalence can be as high as 10%^3^. In most of Sub-Saharan Africa the HBV vaccine is administered in early infancy at 6, 10 and 14 weeks, but pediatric seroprotection proportions and rates are not determined or documented as part of national EPIs monitoring. Additionally, up to 20% of immunocompetent HBV vaccine recipients may fail to respond even to the full three doses^4–7^. For instance, Rey-Cuille et al.^26^ revealed as low as 43% and 58% immune response to HBV vaccine among the under 4-year-olds in Dakar, Senegal in 2007 and 2010 respectively. However, in general the African pediatric HBV vaccine seroprotection remain variably characterized and inconclusive.

Monitoring of the long-term persistence of HBV vaccine responses informs design, review and implementation of effective vaccination programs for adequate control and or elimination of HBV infection. Adoption of, and for timing of booster doses, especially in suboptimal responders may in part be informed by such studies. Even in countries located within the same geographical settings, studies continue to find wildly differing HBV vaccine immune responses and rates of waning. In Tunisia, Ghana and Egypt, studies documented HBV seroprotection of 68.8%, 56% and 8.9% respectively among 17-year-olds^8–10^. The alarmingly low seroprotection proportion in Egypt was accompanied by a recommendation for a booster dose. A recent (2023) review of the Mauritanian Infant Hepatitis B vaccination Program revealed a seroprotective proportion of 93% among those under 2 years, but protection was only 58% and 29% among the 4–5 year, and 10–12 year old children respectively^11^. Therefore, much as contemporary literature reports a 20 year or possibly lifelong seroprotection conferred by HBV vaccination^12–14^, the periodic outbreaks of HBV and the stagnated HBV seroprevalence in Africa justify a need for investigation of proportions of HBV seroprotection in the era of infant vaccination.

Many African countries integrated HBV vaccination onto their national extended programs for Immunization (EPI) by 2002, a 15–17-year age group gives us an opportunity to probe the persistence of HBV seroprotection in this age group as a surrogate for effectiveness of the immunization programs. Fledgling emancipation, combined with peer pressures exposes this age group to behaviors associated with increased HBV infection such as sexual activity, tattooing and sharing of injectable or sharps. It is worth mentioning that populations at heightened risk of infection benefit most from having the sero-protective levels of the HBV vaccine responses. Therefore, if boosting is needed, it should be preferably administered in the adolescent population.

Much as childhood HBV immunization remains fundamental to elimination of HBV, characterization of immune responses, their persistence to late childhood and their determinant factors are crucial to deployment of strategies to elimination of HBV infections. A cumulative population of non-responsive childhood vaccine recipients constitutes a formidable pool of high-risk population with the potential to sustain propagation of HBV infections further, thus eroding gains in regional and global prevention efforts. A meta-analysis to verify the extent of immune protection accorded by the HBV vaccines to African children, and persistence to 17 years is important and informs vaccinologists and programs to deploy optimized interventions prior to later years associated of increased HBV transmission exposures and decreased protective immunity. This meta-analysis provides comprehensive findings on HBV vaccine seroprotection proportions among African children of up to 17 years of age. This will in turn inform public health strategies, including immunization programs targeting reduction of HBV incidence, improving population quality of life, better health and household incomes.

## Results

### Study selection

**Figure 1:**
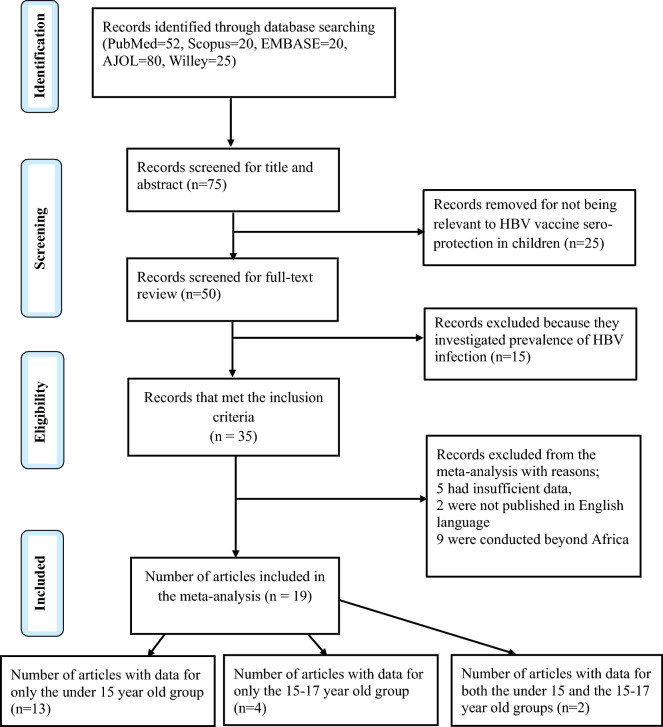
The PRISMA flow chart summarizing the data base searched, screening procedure and the eligible studies for inclusion in data synthesis.

The PRISMA flow chart (Fig. 1) summarizes the method for selection of articles included in this meta-analysis. We initially retrieved 197 records through the primary database searches; PubMed (52), Scopus (20), EMBASE (20), AJOL (80), and Willey library (25). Of the 197 records, 50 records were selected based on relevancy of their titles and abstracts to HBV seroprotection in children. From these, 15 articles were excluded for dwelling on prevalence of HBV infection rather than vaccine seroprotection. Ultimately, 35 articles met the inclusion criteria from which the following studies were removed with reasons; 2 were not published in English, 9 were conducted beyond Africa, and 5 had inaccessible or insufficient data.

**Figure 2:**
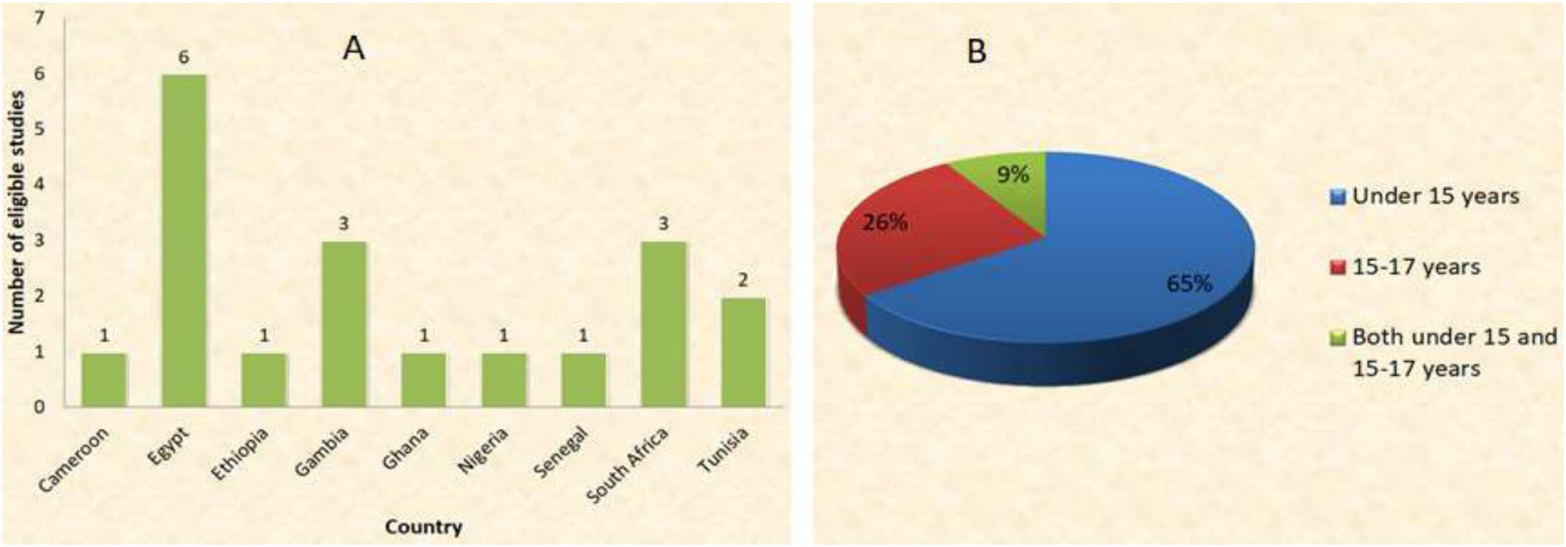
Records eligible for inclusion in the systematic review and meta-analysis; (**A**):—by country, (**B**):—by age.

In total, 19 studies with a total sample size of 10,730 were included in our meta-analysis (Table 1). Of these, 6 studies (31.6%) were from Egypt, 2 (10.5%) from Tunisia, 1 (5.26%) from Nigeria, 1 (5.26%) from Ethiopia, 3 (15.8%) from South Africa, 1 (5.26%) from Ghana, 1 (5.26%) from Senegal, 1 (5.26%) from Cameroon and 3 (15.8%) from Gambia (Fig. 2). Consequently 9 (47.4%), 6 (31.6%), and 3(15.8%) records from North, West and Southern Africa regions were respectively included in the data synthesis. Only a paucity of pediatric seroprotection studies has been published in the East African region. Therefore, one record from the East African region was not included in sub-group analysis. Additionally, there were no identifiable studies from the Southern Africa region for the children 15–17 years of age.

**Table 1:** The characteristics of eligible studies included in the meta-analysis.

First author	Year	Country	Sampling technique	Study design	Method of Analysis	Quality Score	Sample size	Seroprotection rate; %	
								< 15	15–17
Makhlouf^15^	2016	Egypt	Purposive	Cross sectional	MEIA and flow cytometry	9	440	63.9	15.9
Eladawy^10^	2015	Egypt	Purposive	Cross sectional	ELISA	8	225		8.9
Shaaban^16^	2007	Egypt	Consecutive	Cross sectional	ELISA	8	242	39.4	
El Sawy^17^	1999	Egypt	Consecutive	Cross sectional	MEIA	6	180	81.3	
Abushady^18^	2011	Egypt	Purposive	Cross sectional	ELISA	8	700	42.8	
Tfifha^19^	2019	Tunisia	Purposive	Cross sectional	ECLIA	6	180	77.2	
Odusanya^20^	2011	Nigeria	Purposive	Cross sectional, case-controlled, descriptive	Computer processed lab results analyzed by Epi-Info software	8	822	61	
Chaouch^8^	2016	Tunisia	Stratified, two-stage random cluster	Cross sectional	MEIA, and ECLIA	9	1422	75.1	60.8
Teshome^21^	2019	Ethiopia	Multistage probability	Prospective cross sectional	ELISA	8	450	54.3	
Prabdial-Sing^22^	2019	South Africa	Convenience	Cross sectional	ECLIA	7	450	67.3	
Tsebe^23^	2001	South Africa	Purposive	Cross sectional	Imx or Axsym kits (Abbort labs)	8	598	86.8	
Madhi^24^	2019	South Africa	Purposive	Longitudinal	ECLIA	8	567	96.1	
Quaye^9^	2021	Ghana	Purposive	Cross sectional	ELISA	8	350	56	
Van der Sande^25^	2007	Gambia	Purposive/Random	RCT/Cross sectional	Commercial EIA	8	492		31.2
Rey-Cuille^26^	2012	Senegal	Purposive/consecutive	Cross-sectional	Commercial EIA	8	242	92	
Rey-Cuille^26^	2012	Cameroon	Purposive/consecutive	Cross sectional	Commercial EIA	8	242	58	
Whittle^27^	2002	Gambia	Simple random/purposive	Cross sectional	ELISA	8	852		64
Mendy^28^	2013	Gambia	Purposive	Cross sectional	ELISA	8	1276		22.4
Reda^29^	2003	Egypt	Purposive (Systematic/stratified random	Cross sectional	ELISA	8	1000	67

Overall, 15 (65%) records covered children under 15 years of age and 6 (26%) for the children between 15 and 17 years of age with publication years ranging from 1999 to 2021, and 2002 to 2016 respectively. However, 2 (9%) records contained data for both the under 15 and 15–17-year-old children (Figs. 1 and 2). The number of participants in the records included in the meta-analysis was 5,370 and 1,940 participants for the under 15 years and those 15–17 years of age were respectively (Table 1).

Our results showed that children under 15 years of age had a higher proportion of seroprotection to HBV as compared to those 15–17 years of age in Africa. Additionally, children in the Western Africa region had a higher proportion of HBV vaccine seroprotection than their counterparts in the Northern region. The HBV vaccine seroprotection among children in the Southern Africa region is markedly higher than that demonstrated in both the Western and Northern Africa regions (Table 2 and Supplementary Figs. S7, S8, S9, S10, S11, S12, S13 and S14).

**Table 2:** Meta-analyses results of pooled prevalence of HBV seroprotection rates by age, African geographical region and year of scientific paper publication.

Sub groups					
variable	category	No	Prevalence% (95%CI)	*p* value (95%CI)	I 2% (95%CI)
	Age				
Age	Below 15	15	69.07 (60.068–77.395)		98.58 (98.25–98.86)
	15–17	6	32.368 (15.560–51.952)	< 0.0001 (34.2454–39.0847)	99.45 (99.29–99.58)
	Region (under 15)				
Region	South	3	85.221 (66.250–97.162)Ref		98.82 (98.02–99.30)
	North	7	64.242 (52.857–74.866)	< 0.0001 (18.312–23.5317)	98.09 (97.27–98.66)
	West	3	70.566 (43.867–91.267)	< 0.0001 (10.5225–18.9504)	98.44 (97.24–99.12)
	Region (15–17)				
	North	3	26.253 (1.951–64.813)		99.60 (99.42–99.72)
	West	3	38.704 (14.724–66.079)	< 0.0001 (9.7793–15.0838)	99.49 (99.24–99.66)
	Under 15 years				
Year	1999–2012	8	67.421 (54.100–79.445)		98.66 (98.20–99.00)
	2016–2021	7	71.475 (58.482–82.872)	< 0.0013 (1.5894–6.5158)	98.56 (98.00–98.96)
	15–17 years				
	2002–2013	2	4.306 (0.228–13.097)		98.19 (95.88–99.21)
	2015–2016	3	26.253 (1.951–64.813)	< 0.0001 (19.8746–24.03)	99.60 (99.42–99.72)

### Proportion of HBV vaccine seroprotection among children up to 17 years of age

We included 15 records for the children below 15 years of age and 6 records for the children 15–17 years of age in our meta-analysis (Table 2, Supplementary Figs. S1, S2, S3 and S4). Heterogeneity was persistently high (I 2 ≥ 98%, *p* < 0.0001) for all the analyses and hence the random effect model was used to determine prevalence of seroprotection. Both the Egger’s and Begg’s test found no evidence for sources of publication bias (*p* > 0.1). Our results showed a significantly higher (*p* < 0.0001) HBV vaccine seroprotection among children under 15 years of age (69.07%) than children 15–17 years (32.368%) (Table 2, Supplementary Figs. S1, S2, S3 and S4).

### Proportion of HBV vaccine seroprotection among children up to 17 years of age in the Northern, Western, and Southern Africa regions

Our analysis included 3, 7 and 3 records for children under 15 years from the Southern, Northern and Western Africa regions respectively (Table 2, Supplementary Figs. S5, S6, S7, S8, S9 and S10). For children 15–17 years of age, 3 records from the Northern and 3 from the Western Africa regions were included in our analysis. Similarly, the heterogeneity was high for all the studies (I 2 ≥ 98%, *p* < 0.0001). Consequently, a random effect model was utilized to determine the prevalence. Egger’s and Begg’s test were used to assess the bias and, there was no evidence of publication bias (*p* > 0.1). Additionally, the funnel plots in which the standard error of prevalence of each study was plotted against its prevalence, displayed a symmetrical spread of the plots, suggesting little or no evidence of publication bias (Supplementary Figs. S6, S8 and S10). Our results showed that children under 15 years from the Southern Africa region demonstrated significantly higher proportion of HBV seroprotection (85.221%, *p* < 0.0001) than their counterparts in the Northern and Western Africa. The Northern region demonstrated the least proportion of seroprotection (64.242%, *p* < 0.0001) among this age group (Table 2, Supplementary Figs. S7 and S8). Children 15–17 years of age from the Western Africa region demonstrated a low HBV vaccine seroprotection (38.704%, *p* < 0.0001) but was higher than proportions for the Northern Africa region (26.253%, *p* < 0.0001) (Table 2, Supplementary Figs. S11, 12, 13 and 14).

### Proportion of HBV vaccine seroprotection among children up to 17 years of age from 1999 to 2021

We included 15 and 5 studies in our meta-analysis for children under 15 years and children 15–17 years of age respectively. For children under 15 years, 8 records published between 1999 and 2012 were analyzed, while 7 records published between 2016 and 2021 were also analyzed. However, for children 15–17 years, 2 records published between 2002 and 2013 were analyzed, and additionally 3 records published between 2015 and 2016 were also analyzed. All studies used to determine the prevalence illustrated a high heterogeneity (I2 > 98%, *p* < 0.0001) and hence the random effect model was used (Table 2). Our analysis indicated that proportion of HBV vaccine seroprotection was high (67.421% and 71.475 respectively, *p* < 0.0013) (Table 2, Supplementary Figs. S15, S16, S17 and S18) in children under 15 years when comparing studies published between 1999 and 2012 and between 2016 and 2021. However, studies for children 15–17 years published between 2015 and 2016 showed low but significantly higher HBV vaccine seroprotection (26.253%, *p* < 0.0001) than studies published between 2002 and 2013 (4.306%) (Table 2, Supplementary Figs. S19, S20, S21 and S22). Publication bias was evidenced in studies for children 15–17 years published between 2002 and 2013 as demonstrated by the Egger’s (*p* = 0.0001) but not the Begg’s test (*p* = 0.3173). However, there was symmetrical spread of studies from the funnel plot of the standard error of prevalence against its prevalence (Supplementary Fig. S20).

## Discussion

The robustness and duration of seroprotection accorded by hepatitis B vaccine depends on a number of baseline endogenous and exogenous factors such as age, body mass index, sex, concomitant diseases (like HIV infection, diabetes, and chronic liver/kidney disease), psychological stress, nutrition, smoking, alcohol consumption, race, vaccine type, dose and vaccination schedule^30–32^.

Studies have been conducted globally to evaluate the immune protection accorded by HBV vaccine and its persistence among the pediatric population^8–10,12–29,33,34^. However, there is paucity of such studies in Africa. Where such studies have been done, their findings are inconclusive and significantly vary due to the different sample sizes, geographical regions, methods and other study parameters. In this meta-analysis we provide a holistic evaluation of HBV vaccine seroprotection proportions in Africa among childhood recipients, of up to 17 years of age. We have used a pooled sample size of 10,730, significantly improving statistical power that affects other similar studies^10,19,34^.

Here, we report that the proportion of children under 15 years of age with HBV vaccine-specific seroprotection is significantly higher (*p* < 0.0001) (69.07%) than their counterparts 15–17 years of age (32.368%). This finding is in agreement with Egyptian^15^ and Tunisian^8^ studies. Immune protection accorded by HBV vaccine wanes over time, perhaps contributing to the significantly reduced proportion of seroprotection demonstrated in the 15–17 year olds age group^10,15,28^. More so, the 15–17 year olds received the HBV vaccination in the early to mid-2000s; a time when many African countries were just initiating and or rolling out HBV vaccination into their national EPIs. It is possible that challenges in the early phases of HBV vaccine integration and roll-out in the national EPIs affected coverage and completion of doses that in part could explain the lower proportion seen among the 15–17 years old children^10^. What is apparent though is that there is a big pool of children 15–17 years of age who are at increased risk of contracting HBV on exposure yet this age bracket correlates with onset of socio-behavioral activities associated with increased risk to HBV infection. We report that studies among the under 15-year-olds done between 1999–2012 and then from 2016 to 2021, proportions of HBV vaccination seroprotection were very similar (67.421% and 71.475 respectively, *p* < 0.0013), indicating that challenges of implementing a nascent HBV vaccination program, if any, did not significantly impact on proportions of children with induced seroprotection. A number of studies^12–14,35^ have also reported relatively high proportions of seroprotection for children under 5 years. The United Nations Sustainable Development Goals aim to eliminate viral hepatitis by the year 2030^36^, and the WHO-UNICEF, continues to advocate for improved vaccination coverage and proportions of seroprotection that meet the herd immunity threshold. The results above indicate a concerted effort for African national EPIs to attain the desired coverage right from the start of HBV integration in childhood vaccination programs.

Regardless of the age groups, we generally are seeing low proportions of HBV vaccine seroprotection in much of Africa. We have found studies reporting even lower proportions of seroprotection (39.4% and 42.8%) among Egyptian children under 15 years^16,18^. These reduced proportions of seroprotection in HBV vaccination are contrary to those reported in Western developed countries. Studies conducted in higher income countries other than Africa that report significantly higher proportions of seroprotection among adolescent children. Probably the profoundly reduced HBV infection rates in such countries are attributable to higher seroprotection rates compared to Africa. A study that assessed the immune persistence conferred by a Chinese hamster ovary (CHO)-derived hepatitis B vaccine (HepB) 17–20 years after primary immunization during early life, demonstrated seroprotection rates of 74.5% among the participants^13^. Similarly a study conducted in Germany confirmed that paediatric doses of hepatitis B vaccine confer long-term protection against hepatitis B in adolescents up to 15–16 years; with pre-booster dose seroprotection rates of 65.4%, and produce robust immune memory^37^. Reduced sero protective levels in Africa have been attributed to several factors. Waning of immunity over time, often measured as reduction or loss of detectable levels of anti-HBs antibody titers has been cited by many^15,16,21,22^. Evidence for waning responses is supported by our results showing that articles published between 1999 and 2012, and those published between 2016 and 2021 show relatively high and comparable proportions of seroprotection (67.421% and 71.475 respectively, *p* < 0.0013) among children who received the HBV vaccine less than 15 years ago while the reverse is true in those 15–17 year olds. The correlation of antibodies with seroprotection is driven by the belief that neutralizing antibodies are the major correlate of protection against HBV infection, and several infectious agents^38–41^. However, another side of the argument is that levels of anti-HBs neutralizing antibodies may not accurately correlate with seroprotection because recall or anamnestic responses may ensure effective protection against HBV upon exposure^13,35,42–45^. In this context, the waning of seroprotection may not be of clinical or epidemiological consequence. Also, the type of vaccination schedule adopted has been associated with variations in proportions and magnitude of seroprotection induced. Some national EPIs in Africa were subscribing to the 2, 4 and 6 months schedule^8,10,15,16,29^ while others were implementing the 6, 10 and 14 weeks schedule^9,20,22,27,46^. However, recently many national EPIs have moved to a modified version of the later schedule that starts with a HBV vaccine shot given at birth that is now thought to be more effective^45,47–49^. Another source of reduced proportions of seroprotection in Africa has been the plethora of current, past and future exposures to several infectious agents. Particularly, parasitic infections such as *Schistosoma mansoni* and *Plasmodium falciparum* have been associated with blunted responses to vaccines^38,40,50,51^, including to HBV vaccination^39^.

Even within Africa we report seeing variations in proportions of HBV vaccine seroprotection. For instance, children under 15 years from the Southern Africa region demonstrate significantly higher HBV vaccine seroprotection proportions (85.221%, *p* < 0.0001) than their counterparts in the Northern and Western Africa. Equally, children 15–17 years of age from the Western Africa region demonstrate higher HBV vaccine seroprotection proportion (38.704%, *p* < 0.0001) than those from the Northern Africa region (26.253%, *p* < 0.0001).Northern Africa demonstrates the least proportions in seroprotection (64.242%, *p* < 0.0001) among this age group^8–10,15–20,22–29^. A closer look at the HBV vaccine schedule implemented by EPIs in Southern and Western Africa regions shows that it has been the 6, 10, and 14 weeks schedule^9,23,26^ that is associated with better seroprotective responses. Northern Africa EPIs on the other hand have been implementing the less effective 2, 4, and 6 months schedule^8,52^. Sub-optimal or outright non-response to HBV vaccination also has a genetic dimension. Contemporary literature continues to indicate that individuals with a genetic predisposition for non-response to HBV vaccine may have a defect in their Human Leukocyte Antigen (HLA) and T-cell receptor (TCR) and hence dysfunctional antigen presentation and stimulation of T Helper cells respectively^32^. Certain HLA types are associated with increased or reduced antibody response to hepatitis B vaccines in different individuals^53^. To the best of our knowledge, such studies have not been conducted to conclusion in Africa, yet the African people and generally people of the black race are known to be the most genetically diverse^54–57^. Inherent heterogeneity therein can significantly determine the variations in proportions of HBV sero protection among children under 15 years in the Southern, Western and Northern Africa.

We also indicate here that studies for children 15–17 years which were published between 2015 and 2016 showed low but significantly higher proportions of HBV vaccine seroprotection (26.253%,%, *p* < 0.0001) than studies published between 2002 and 2013 (4.306%). Here we report publication bias in studies for children 15 to 17 years published between 2002 and 2013 as demonstrated by the Egger’s (*p* = 0.0001) but not the Begg’s test (*p* = 0.3173). These two studies were conducted in the same country; Gambia, and done in 2002 and 2013 respectively, hence probably vindicating the noted bias. However, there was symmetrical spread of studies from the funnel plot of the standard error of prevalence against its prevalence (Supplementary Fig. S20). As earlier noted, the 15–17-year age group is a high risk cohort for HBV and other sexually transmitted diseases yet it is demonstrating lower HBV vaccine seroprotection. However, studies published between 2015 and 2016 were probably conducted on children 15–17 years of age who were immunized in the HBV vaccination era^2^, and after effective integration of HBV vaccines on EPIs of all countries (1997)^58^. Studies published between 2002 and 2013 were probably conducted on 15–17-year-old children who were ineffectively and inadequately vaccinated in the pre-vaccination era, and perhaps markedly limiting the HBV seroprotection rates.

This meta-analysis establishes proportions of HBV seroprotection among children up to 17 years of age in Africa. Children under 15 years of age demonstrate a significantly higher proportion of HBV vaccine seroprotection than those 15–17 years of age. The proportion of HBV vaccine seroprotection in children under 15 years are relatively high with the Southern Africa region demonstrating significantly higher proportion than its counterparts in the Northern and Western Africa. Notably, African children 15–17 years of age generally present with reduced proportion of HBV vaccine seroprotection. We further demonstrate generally higher proportions of HBV vaccine seroprotection in children under 5 years reported by studies conducted between 1999 and 2012, and those published between 2016 and 2021. However, studies among children 15–17 years published between 2015 and 2016 show low proportions of HBV vaccine seroprotection, although higher than proportions reported by studies published between 2002 and 2013. Whereas successful integration of the efficacious HBV vaccine on the EPIs has been a major achievement in the reduction of HBV infection in Africa, this meta-analysis indicates profoundly reduced HBV vaccine seroprotection rates among current adolescents of 15–17 years of age. Systematically designed EPI evaluation studies may be required to supplement and augment our findings, and perhaps establish and clarify the need for booster dose vaccination, particularly in the most at risk pediatric African populations.

We acknowledge some limitations in our study. Limited number of EPI monitoring and evaluation reports and publications on HBV vaccine seroprotection in Africa may have limited geographical coverage or representations of the meta-data used, particularly the East Africa regions. We could not confirm if all the study participants enrolled in the primary studies had completed all the WHO recommended three HBV vaccine doses. This could certainly have led to an underestimation of the seroprotective proportions reported in the primary, and hence in this study. To mitigate both of the limitations above, we recommend meta-analyses that consider only the better designed national EPI evaluation data as they become more available. We considered no genetic diversity across the African continent in explaining the regional variability in sero-proportions observed. Therefore, the differences in seroprotection observed across the African regions may be due to genetics rather than vaccination strategy. All these study limitations notwithstanding, we pooled the prevalence of seroprotection to commendably enhance the representativeness and power of our meta-analysis.

## Methods

### Systematic review protocol registration, information sources, and search strategies

This study sought to determine the proportions of immune protection against hepatitis B vaccine among HBV vaccine childhood recipients of under 15, and 15–17 years in Africa. The study protocol was submitted for registration with the International Prospective Register of Systematic Reviews (PROSPERO), University of York Centre for Reviews and Dissemination (https://www.crd.york.ac.uk/PROSPERO). The study required no ethical review to be conducted. The findings of the review were reported based on the Preferred Reporting Items for Systematic Review and Meta-Analysis (PRISMA) 2020 statement checklist^59^. Our search scope included electronic databases; African Journals Online (AJOL), EMBASE, Willey library, Scopus, and PubMed, for studies published from 1999 to 2021 that investigated the seroprotection accorded by hepatitis B vaccine among childhood recipients under 15, and 15–17 years in Africa.

Weekly reviewers’ evaluation meetings were conducted for detailed review of accessible full journal articles, titles and abstracts. Resolution of disagreements among the reviewers (FM, HMK, and BSB) was done by consensus. Only qualified full text articles were retained for data extraction after explicit interrogation by FM, HMK, and BSB to identify duplicate records and other anomalies.

We utilized the population, comparison and outcome (PCO) model as a search strategy. All studies assessing seroprotection to HBV studies that used study participants from Northern, Western, and Southern Africa regions were searched for data extraction. For comparison, the data on the prevalence of seroprotection to HBV infection were searched and then compared among children below 15 years and those between 15 and 17 years. The key outcome was sero-protective level of immune responses to HBV vaccine in the population, while the supplementary outcomes were the relative sero-protective proportions basing on the diagnostic methods used for anti-HBs antibody detection (ELISA, EIA, ECLIA, and MEIA) and factors associated with the sero-protective levels.

The search terms used are presented in Supplementary Information File, Table S1. These were used either in isolation or in combination using the Boolean operators ‘OR’ and ‘AND’. The titles and abstracts were searched for the keywords related to HBV vaccine seroprotection proportion or rate while the full text of each article was searched for the keywords related to protective anti-HBs titers and associated risk factors. The three authors (FM, HMK, and BSB) separately extracted the following data: first author, year of publication, country, sampling technique, study design, sample size, method of detection and quantification of anti-HBs antibodies, and seroprotection rate (Table 1).

### Eligibility criteria and study selection

Records were included in the systematic review and meta-analysis if they were full text, cross sectional or longitudinal study designs determining sero-protective anti-HBs titers (≥ 10 mIU/ml), conducted in Northern, Western, or Southern Africa, and published in English in peer reviewed journals between January of 1999 and December, 2021. We excluded case reports; reviews; conference abstracts; studies with insufficient or inaccessible data; pre-prints; studies with a sample size < 100; studies that never described their sampling technique; studies that investigated seroprotection against other types of viral hepatitis (A, C, D, or E); studies in languages other than English; and studies published before January, 1999 or after December, 2021 (Table 1).

### Quality assessment and data management

The Newcastle–Ottawa Scale (NOS) was used to assess quality^60^. The three dimensions of selection, comparability and exposure were considered, as described in the scale. Articles were assigned one point for each attribute they adequately addressed within the above-mentioned dimensions. For this meta-analysis, the selection assessed the representativeness of the sample, the sampling technique, the sample size, the inclusion criteria, and the scope of the study. Comparability assessed the inclusion of seroprotection prevalence in the primary studies and compared them between vaccine recipients below 15 years and those 15–17 years. Sero -protection proportions across the Northern, Western, and Southern regions of Africa were also compared. Assessment of inclusion of the exposure in the primary studies was done by looking at the outcome of the study, and a statistical tool used for the analysis (Supplementary Information File, Table S2). According to the scale, studies with scores of 9–8 were considered very high quality; 7–6, high quality; and 5–4, moderate quality. Those with scores ≤ 3 were considered unsatisfactory and were rejected. The three reviewers (FM, HMK, and BSB) independently assessed the articles for their overall methodological quality. The authors individually participated in daily entry of data derived from research papers, into the spreadsheet. The authors additionally removed duplicate papers after comparing their archives. During the review process (screening, eligibility, and inclusion in meta-analysis), the primary studies were independently assessed by three hepatitis B virus specialists (FM, HMK, and BSB). From each study, the following data were extracted: first author, year of publication, country, sampling technique, sample size, quality score (QS), method of detection and quantification of anti-HBs antibodies, and seroprotection rate.

### Risk of bias in individual studies

To subdue the effects of selection bias, we reviewed the data collection procedures in the primary studies (retrospective or prospective), study design (cohort, cross-sectional, or case control), and recruitment strategy (from the community or from the hospital). For the information bias, we extracted information on methods used to detect and quantify anti-HBs antibodies (ELISA, ELISA, EIA, ECLIA, MEIA and other immune assays). When more than one method was used to detect and quantify anti-HBs antibodies, data were extracted using the more accurate method, as indicated by the evaluator. All studies with a sample size less than 100 were excluded to avoid biasing the results.

### Publication bias and data synthesis

The Begg’s test, which uses Kendall’s rank correlation coefficient between the meta-analysis effect size and the study weight, was used to assess publication bias^61^. Funnel plots further verified the existence of overall publication bias. The Begg’s test determined funnel plot asymmetry between the effect size and the meta-analysis study weight. In this test, *p* > 0.05 signified a lack of publication bias (not significant). The test I2 statistic was used to assess heterogeneity among studies (Table 2). Sources of heterogeneity were assessed by sub-group analysis, sensitivity analysis, and meta-regression. Pooled proportions, test of proportions, relative risk (RR), and the corresponding 95% CI were used to assess seroprotection rates various geographical regions. As a result of high heterogeneities (I2 > 73% and *p* het < 0.05), the random-effects model (REM) was used to pool the seroprotection rates. All the calculations were done using the Medcalc software version 19.1.3. Seroprotection rates and its associated factors, and heterogeneity analyses among studies were done at 95% CI, and a *p* < 0.05 was considered significant. Forest plots were used to graphically present representative results (Supplementary Information File, Figs. S1, S3, S5, S7, S9, S11, S13, S15, S17, S19 and S21). The seroprotection rate in each study in the forest plot is indicated by a blue square. The size of the square represents the weight contributed by each study in the meta-analysis. The pooled seroprotection rate for the REM is shown by the blue diamond.

### Supplementary Information

Supplementary Figure S1.

Supplementary Figure S2.

Supplementary Figure S3.

Supplementary Figure S4.

Supplementary Figure S5.

Supplementary Figure S6.

Supplementary Figure S7.

Supplementary Figure S8.

Supplementary Figure S9.

Supplementary Figure S10.

Supplementary Figure S11.

Supplementary Figure S12.

Supplementary Figure S13.

Supplementary Figure S14.

Supplementary Figure S15.

Supplementary Figure S16.

Supplementary Figure S17.

Supplementary Figure S18.

Supplementary Figure S19.

Supplementary Figure S20.

Supplementary Figure S21.

Supplementary Figure S22.

Supplementary Table S1.

Supplementary Information.

## Data Availability

All data generated or analyzed during this study are included in this published article.

## Abbreviations

AJOL: African Journal Online
Anti-HBs: Antibody against the hepatitis B surface antigen
EIA: Enzyme immuno assay
ECLIA: Electrochemiluminiscence immunoassay
ELISA: Enzyme linked immunosorbent assay
EPI: Extended program on immunization
FEM: Fixed effect model
HBsAg: Hepatitis B surface antigen
HBV: Hepatitis B Virus
MEIA: Micro-particle enzyme immuno assay
NOS: Newcastle–Ottawa scale
PRISMA: Preferred reporting items for systematic review and meta-analysis
QS: Quality assessment
REM: Random effect model
WHO: World Health Organization
