# Supplementary figures and images for: A systematic review reveals that African children of 15–17 years demonstrate low hepatitis B vaccine seroprotection rates
Source: Sci Rep. 2023 Dec 13;13:22182. doi: 10.1038/s41598-023-49674-1 (PMC10719251; doi:10.1038/s41598-023-49674-1)

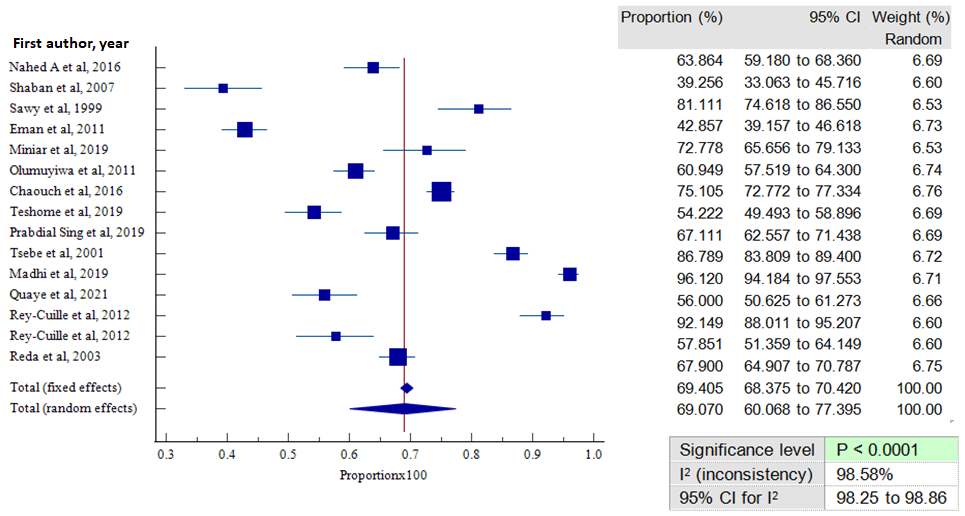


**Fig. S1.** Forest plot showing HBV vaccine sero-protection rate among children under 15 years of age

Supplement: Supplementary file 1 — Supplementary Figure S1. [file 41598_2023_49674_MOESM1_ESM.docx]

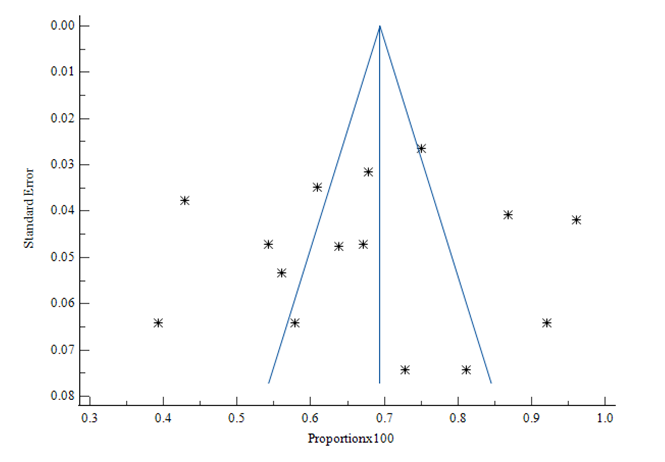


**Fig. S2.** Funnel plot to assess publication bias in studies of children under 15 years of age

Supplement: Supplementary file 2 — Supplementary Figure S2. [file 41598_2023_49674_MOESM2_ESM.docx]

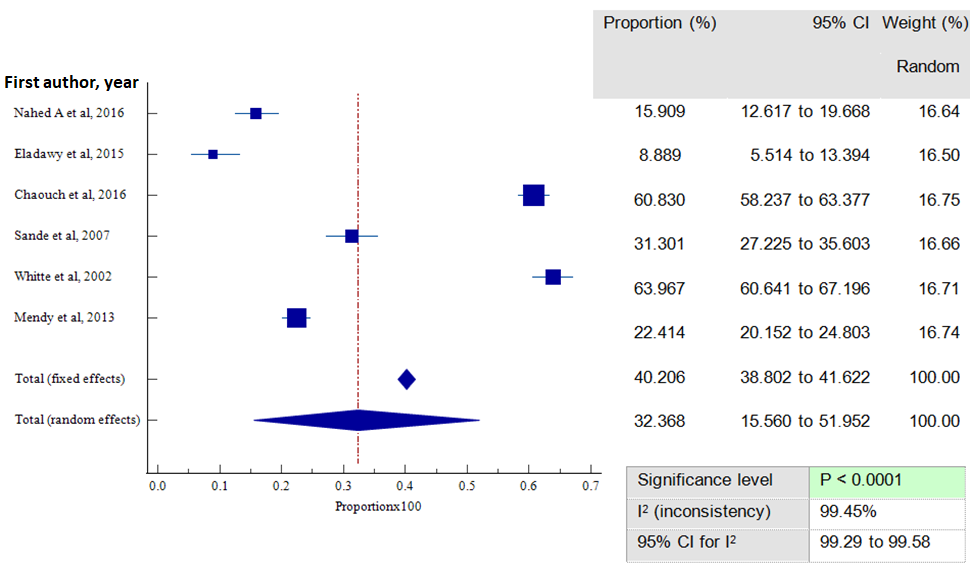


**Fig. S3.** Forest plot showingHBV vaccine sero-protection rate among children under 15 to 17 years of age

Supplement: Supplementary file 3 — Supplementary Figure S3. [file 41598_2023_49674_MOESM3_ESM.docx]

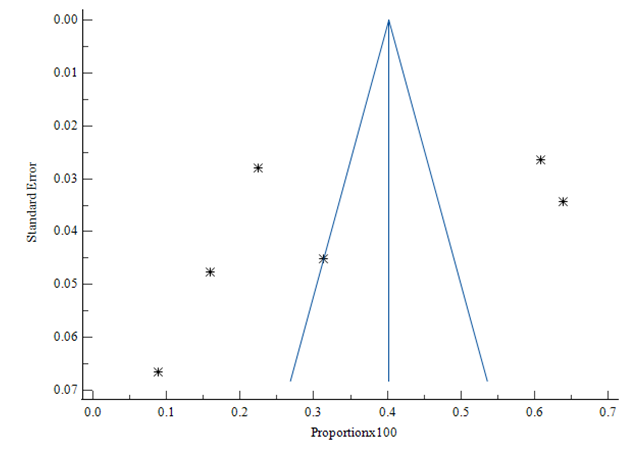


**Fig. S4.** Funnel plot to assess publication bias in studies of children 15 to 17 years of age

Supplement: Supplementary file 4 — Supplementary Figure S4. [file 41598_2023_49674_MOESM4_ESM.docx]
