# Supplementary material for: A systematic review reveals that African children of 15–17 years demonstrate low hepatitis B vaccine seroprotection rates
Source: Sci Rep. 2023 Dec 13;13:22182. doi: 10.1038/s41598-023-49674-1 (PMC10719251; doi:10.1038/s41598-023-49674-1)
Supplement: Supplementary file 16 — Supplementary Figure S16. [file 41598_2023_49674_MOESM16_ESM.docx]

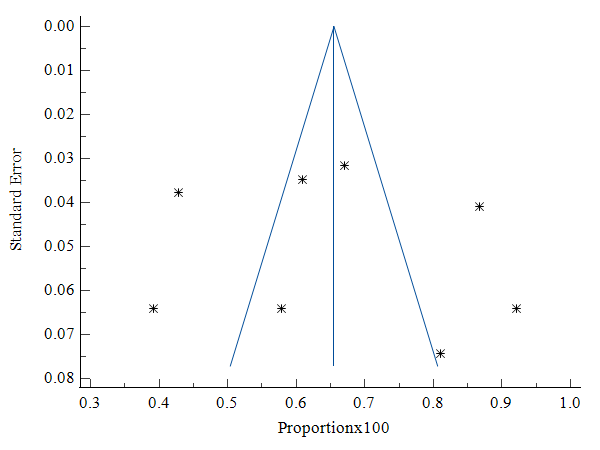


**Fig. S16.** Funnel plot to assess publication bias in scientific articles published from 1999 to 2012 and investigating children under 15 to years of age.
