# Supplementary material for: A systematic review reveals that African children of 15–17 years demonstrate low hepatitis B vaccine seroprotection rates
Source: Sci Rep. 2023 Dec 13;13:22182. doi: 10.1038/s41598-023-49674-1 (PMC10719251; doi:10.1038/s41598-023-49674-1)
Supplement: Supplementary file 20 — Supplementary Figure S20. [file 41598_2023_49674_MOESM20_ESM.docx]

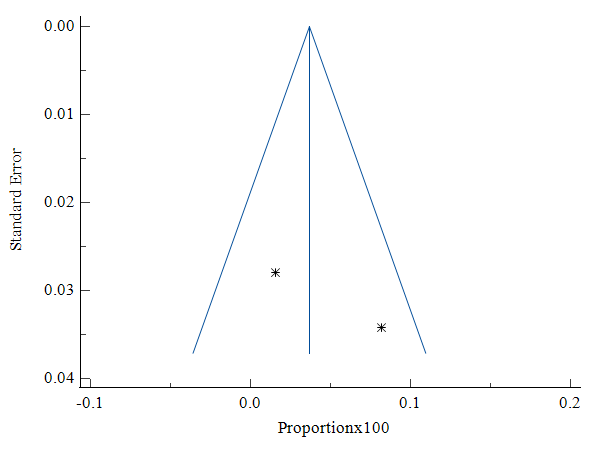


**Fig. S20.** Funnel plot to assess publication bias in scientific articles published from 2002 to 2013 and investigating children 15 to 17 years of age.
