# Supplementary material for: A systematic review reveals that African children of 15–17 years demonstrate low hepatitis B vaccine seroprotection rates
Source: Sci Rep. 2023 Dec 13;13:22182. doi: 10.1038/s41598-023-49674-1 (PMC10719251; doi:10.1038/s41598-023-49674-1)
Supplement: Supplementary file 21 — Supplementary Figure S21. [file 41598_2023_49674_MOESM21_ESM.docx]

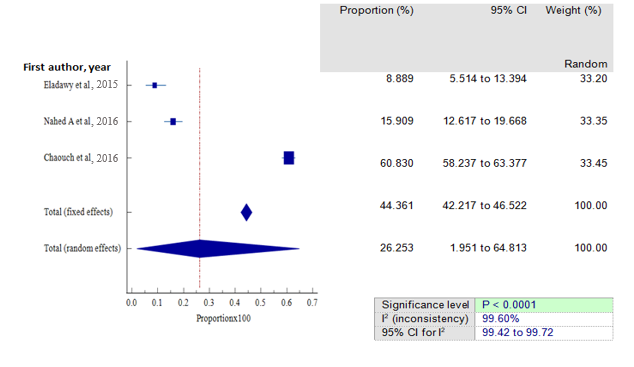


**Fig. S21.** Forest plot showing HBV vaccine sero-protection rate in scientific articles published from 2015 to 2016 and investigating children 15 to 17 years of age.
