# Supplementary material for: A systematic review reveals that African children of 15–17 years demonstrate low hepatitis B vaccine seroprotection rates
Source: Sci Rep. 2023 Dec 13;13:22182. doi: 10.1038/s41598-023-49674-1 (PMC10719251; doi:10.1038/s41598-023-49674-1)
Supplement: Supplementary file 23 — Supplementary Table S1. [file 41598_2023_49674_MOESM23_ESM.docx]

**A systematic review and meta-analysis reveals that African children 15 to 17 years of age demonstrate significantly reduced hepatitis B vaccine sero-protection rates**

Author List; Fahad Muwanda, Hakim Sendagire, Gerald Mboowa, David Patrick Kateete, Beatrice Achan, Ezekiel Mupere, Hussein Mukasa Kafeero_,_ and Bernard Ssentalo Bagaya

* Correspondence: [muwandafahad@gmail.com](mailto:muwandafahad@gmail.com)

**Table S1.** Keywords used for searching in databases

| **Field searched** | **Key words** |
| --- | --- |
| HBV vaccine sero-protection by age | Anti-HBs antibody, hepatitis B vaccine, sero-protection, children, North Africa, West Africa, South Africa, East Africa. |
| HBV vaccine sero-protection by geographical region | Anti-HBs antibody, hepatitis B vaccine, sero-protection, North Africa, West Africa, South Africa, East Africa. |
