# Supplementary material for: A systematic review reveals that African children of 15–17 years demonstrate low hepatitis B vaccine seroprotection rates
Source: Sci Rep. 2023 Dec 13;13:22182. doi: 10.1038/s41598-023-49674-1 (PMC10719251; doi:10.1038/s41598-023-49674-1)
Supplement: Supplementary file 24 — Supplementary Information. [file 41598_2023_49674_MOESM24_ESM.docx]

Table S2. Detailed Newcastle-Ottawa Scale for each included study in the meta-analysis.

| Study | Selection | | | | | Comparability | | Exposure | | Q S |
| --- | --- | --- | --- | --- | --- | --- | --- | --- | --- | --- |
|  | Representativeness of the sample | Sampling technique | Sample size | Inclusion criteria stated | Scope of the study defined | Assessed for sero-protection | Compared HBV sero-protection among study groups | Assessment of outcome | Statistical test |  |
| ([Makhlouf et al., 2016](#_ENREF_6)) | 1 | 1 | 1 | 1 | 1 | 1 | 1 | 1 | 1 | 9 |
| ([Eladawy, Gamal, Fouad, & El-Faramawy, 2015](#_ENREF_4)) | 1 | 1 | 1 | 1 | 1 | 1 | 0 | 1 | 1 | 8 |
| ([Shaaban, Hassanin, Samy, Salama, & Said, 2007](#_ENREF_13)) | 1 | 1 | 1 | 1 | 1 | 1 | 0 | 1 | 1 | 8 |
| ([El Sawy & Mohamed, 1999](#_ENREF_3)) | 0 | 1 | 0 | 1 | 1 | 1 | 0 | 1 | 1 | 6 |
| ([Abushady et al., 2011](#_ENREF_1)) | 1 | 1 | 1 | 1 | 1 | 1 | 0 | 1 | 1 | 8 |
| ([Tfifha et al., 2019](#_ENREF_15)) | 0 | 1 | 0 | 1 | 1 | 1 | 0 | 1 | 1 | 6 |
| ([Odusanya, Alufohai, Meurice, & Ahonkhai, 2011](#_ENREF_8)) | 1 | 1 | 1 | 1 | 1 | 1 | 0 | 1 | 1 | 8 |
| ([Chaouch et al., 2016](#_ENREF_2)) | 1 | 1 | 1 | 1 | 1 | 1 | 1 | 1 | 1 | 9 |
| ([Teshome et al., 2019](#_ENREF_14)) | 1 | 1 | 1 | 1 | 1 | 1 | 0 | 1 | 1 | 8 |
| ([Prabdial-Sing et al., 2019](#_ENREF_9)) | 1 | 0 | 1 | 1 | 1 | 1 | 0 | 1 | 1 | 7 |
| ([Tsebe et al., 2001](#_ENREF_16)) | 1 | 1 | 1 | 1 | 1 | 1 | 0 | 1 | 1 | 8 |
| ([Madhi et al., 2019](#_ENREF_5)) | 1 | 1 | 1 | 1 | 1 | 1 | 0 | 1 | 1 | 8 |
| ([Quaye, Narkwa, Domfeh, Kattah, & Mutocheluh, 2021](#_ENREF_10)) | 1 | 1 | 1 | 1 | 1 | 1 | 0 | 1 | 1 | 8 |
| ([Van der Sande et al., 2007](#_ENREF_17)) | 1 | 1 | 1 | 1 | 1 | 1 | 0 | 1 | 1 | 8 |
| ([Rey-Cuille et al., 2012](#_ENREF_12)) | 1 | 1 | 1 | 1 | 1 | 1 | 0 | 1 | 1 | 8 |
| ([Rey-Cuille et al., 2012](#_ENREF_12)) | 1 | 1 | 1 | 1 | 1 | 1 | 0 | 1 | 1 | 8 |
| ([Whittle et al., 2002](#_ENREF_18)) | 1 | 1 | 1 | 1 | 1 | 1 | 0 | 1 | 1 | 8 |
| ([Mendy et al., 2013](#_ENREF_7)) | 1 | 1 | 1 | 1 | 1 | 1 | 0 | 1 | 1 | 8 |
| ([Reda et al., 2003](#_ENREF_11)) | 1 | 1 | 1 | 1 | 1 | 1 | 0 | 1 | 1 | 8 |

*For quality assessment before inclusion, studies were assigned 1 point if they adequately addressed the following attributes; representativeness of the sample, sampling technique, sample size, inclusion criteria, study scope, sero-protection rates, comparison, outcome, and statistical test as illustrated in the scale above. Studies with scores of 9–8 were considered very high quality; 7–6, high quality; and 5–4, moderate quality. Those with scores ≤3 were considered unsatisfactory and were not included in the meta-analysis.

**References**

Abushady, E. A., Gameel, M. M., Klena, J. D., Ahmed, S. F., Abdel-Wahab, K. S., & Fahmy, S. M. (2011). HBV vaccine efficacy and detection and genotyping of vaccinee asymptomatic breakthrough HBV infection in Egypt. *World journal of hepatology, 3*(6), 147.

Chaouch, H., Hachfi, W., Fodha, I., Kallala, O., Saadi, S., Bousaadia, A., . . . Trabelsi, A. (2016). Impact and long-term protection of hepatitis B vaccination: 17 years after universal hepatitis B vaccination in Tunisia. *Epidemiology & Infection, 144*(16), 3365-3375.

El Sawy, I., & Mohamed, O. (1999). Long-term immunogenicity and efficacy of a recombinant hepatitis B vaccine in Egyptian children. *EMHJ-Eastern Mediterranean Health Journal, 5 (5), 922-932, 1999*.

Eladawy, M., Gamal, A., Fouad, A., & El-Faramawy, A. (2015). Hepatitis B Virus Vaccine immune response in Egyptian children 15-17 years after primary immunization; should we provide a booster dose? *Egyptian Journal of Pediatric Allergy and Immunology (The), 13*(2), 45-48.

Madhi, S. A., López, P., Zambrano, B., Jordanov, E., B’Chir, S., Noriega, F., & Feroldi, E. (2019). Antibody persistence in pre-school children after hexavalent vaccine infant primary and booster administration. *Human vaccines & immunotherapeutics, 15*(3), 658-668.

Makhlouf, N. A., Farghaly, A. M., Zaky, S., Rashed, H. A. G., Abu Faddan, N. H., Sayed, D., . . . El‐Sayed, Y. (2016). The efficacy of hepatitis B vaccination program in upper Egypt: Flow cytometry and the evaluation of long term immunogenicity. *Journal of medical virology, 88*(9), 1567-1575.

Mendy, M., Peterson, I., Hossin, S., Peto, T., Jobarteh, M. L., Jeng-Barry, A., . . . Hall, A. J. (2013). Observational study of vaccine efficacy 24 years after the start of hepatitis B vaccination in two Gambian villages: no need for a booster dose. *PloS one, 8*(3), e58029.

Odusanya, O. O., Alufohai, E., Meurice, F. P., & Ahonkhai, V. I. (2011). Five-year post vaccination efficacy of hepatitis B vaccine in rural Nigeria. *Human vaccines, 7*(6), 625-629.

Prabdial-Sing, N., Makhathini, L., Smit, S. B., Manamela, M. J., Motaze, N. V., Cohen, C., & Suchard, M. S. (2019). Hepatitis B sero-prevalence in children under 15 years of age in South Africa using residual samples from community-based febrile rash surveillance. *PloS one, 14*(5), e0217415.

Quaye, T., Narkwa, P. W., Domfeh, S. A., Kattah, G., & Mutocheluh, M. (2021). Immunosurveillance and molecular detection of hepatitis B virus infection amongst vaccinated children in the West Gonja District in Savanna Region of Ghana. *PloS one, 16*(9), e0257103.

Reda, A., Arafa, M., Youssry, A., Wandan, E., Ab de Ati, M., & Daebees, H. (2003). Epidemiologic evaluation of the immunity against hepatitis B in Alexandria, Egypt. *European journal of epidemiology, 18*(10), 1007-1011.

Rey-Cuille, M.-A., Seck, A., Njouom, R., Chartier, L., Sow, H. D., Ka, A. S., . . . Unal, G. (2012). Low immune response to hepatitis B vaccine among children in Dakar, Senegal. *PloS one, 7*(5), e38153.

Shaaban, F., Hassanin, A., Samy, S., Salama, S., & Said, Z. (2007). Long-term immunity to hepatitis B among a sample of fully vaccinated children in Cairo, Egypt. *EMHJ-Eastern Mediterranean Health Journal, 13 (4), 750-757, 2007*.

Teshome, S., Biazin, H., Tarekegne, A., Abebe, T., Bekele, F., Mihret, A., . . . Howe, R. (2019). Antibody response against hepatitis B virus after vaccination and seroprevalence of HBV in children in Addis Ababa, Ethiopia. *Ethiop Med J*.

Tfifha, M., Kacem, S., Naija, S., Boujaafar, N., Abroug, S., & Trabelsi, A. (2019). Evaluation of antibody persistence after a four-dose primary hepatitis B vaccination and anamnestic immune response in children under 6 years. *Journal of Medical Microbiology, 68*(11), 1686-1693.

Tsebe, K. V., Burnett, R. J., Hlungwani, N. P., Sibara, M. M., Venter, P. A., & Mphahlele, M. J. (2001). The first five years of universal hepatitis B vaccination in South Africa: evidence for elimination of HBsAg carriage in under 5-year-olds. *Vaccine, 19*(28-29), 3919-3926.

Van der Sande, M. A., Waight, P. A., Mendy, M., Zaman, S., Kaye, S., Sam, O., . . . Hall, A. J. (2007). Long-term protection against HBV chronic carriage of Gambian adolescents vaccinated in infancy and immune response in HBV booster trial in adolescence. *PloS one, 2*(8), e753.

Whittle, H., Jaffar, S., Wansbrough, M., Mendy, M., Dumpis, U., Collinson, A., & Hall, A. (2002). Observational study of vaccine efficacy 14 years after trial of hepatitis B vaccination in Gambian children. *Bmj, 325*(7364), 569.
